# 2-(4-Formyl-2,6-dimeth­oxy­phenoxy)­acetic acid

**DOI:** 10.1107/S1600536810043990

**Published:** 2010-10-31

**Authors:** Alain Collas, Christophe M. L. Vande Velde, Frank Blockhuys

**Affiliations:** a Department of Chemistry, University of Antwerp, Universiteitsplein 1, B-2610 Wilrijk, Belgium

## Abstract

In the title compound, C_11_H_12_O_6_, the aldehyde group is disordered over two sites in a 0.79:0.21 ratio. The carb­oxy­lic acid chain is found in the [*ap*,*ap*] conformation due to two intramolecular O—H⋯O  hydrogen bonds.

## Related literature

For related acetic acids substituted in the α-position, see: Lundquist *et al.* (1987 ▶). For conformational and geometric considerations of carb­oxy­lic acids, see: Lide (1964 ▶); Leiserowitz (1976 ▶). For applications of PPV oligomers, see: Chemla (1987 ▶); Bandyopadhyay & Pal (2003 ▶). For hydrogen bonding and crystal engineering, see: Desiraju (1997 ▶); Steiner (2002 ▶).
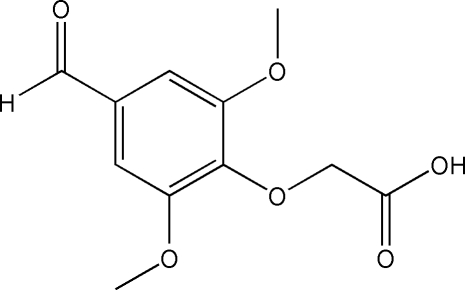


## Experimental

### 

#### Crystal data

C_11_H_12_O_6_
*M* *_r_* = 240.21
Monoclinic, 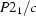

*a* = 9.350 (2) Å
*b* = 7.416 (1) Å
*c* = 17.374 (8) Å
β = 113.67 (3)°
*V* = 1103.4 (6) Å^3^
*Z* = 4
Mo *K*α radiation
μ = 0.12 mm^−1^
*T* = 293 K
0.27 × 0.24 × 0.15 mm

#### Data collection

Enraf–Nonius MACH3 diffractometer
4025 measured reflections
2015 independent reflections
1292 reflections with *I* > 2σ(*I*)
*R*_int_ = 0.030
3 standard reflections every 60 min  intensity decay: 1%

#### Refinement

*R*[*F* ^2^ > 2σ(*F* ^2^)] = 0.036
*wR*(*F* ^2^) = 0.093
*S* = 1.01
2015 reflections
171 parameters
8 restraints
H-atom parameters constrained
Δρ_max_ = 0.15 e Å^−3^
Δρ_min_ = −0.15 e Å^−3^

### 

Data collection: *CAD-4 EXPRESS* (Enraf–Nonius, 1994 ▶); cell refinement: *CAD-4 EXPRESS*; data reduction: *XCAD4* (Harms & Wocadlo, 1996 ▶); program(s) used to solve structure: *SHELXS97* (Sheldrick, 2008 ▶); program(s) used to refine structure: *SHELXL97* (Sheldrick, 2008 ▶); molecular graphics: *ORTEP-3 for Windows* (Farrugia, 1997 ▶); software used to prepare material for publication: *WinGX* (Farrugia, 1999 ▶).

## Supplementary Material

Crystal structure: contains datablocks I, global. DOI: 10.1107/S1600536810043990/zl2319sup1.cif

Structure factors: contains datablocks I. DOI: 10.1107/S1600536810043990/zl2319Isup2.hkl

Additional supplementary materials:  crystallographic information; 3D view; checkCIF report

## Figures and Tables

**Table 1:** Hydrogen-bond geometry (Å, °)

*D*—H⋯*A*	*D*—H	H⋯*A*	*D*⋯*A*	*D*—H⋯*A*
O42—H42⋯O4	0.82	2.11	2.617 (2)	120
O42—H42⋯O5	0.82	2.18	2.932 (2)	153
C6—H6⋯O41^i^	0.93	2.54	3.468 (3)	176
C41—H41*A*⋯O11*B*^ii^	0.97	2.71	3.189 (9)	111
C51—H51*B*⋯O11*A*^iii^	0.96	2.57	3.369 (3)	141
C31—H31*B*⋯O11*A*^iv^	0.96	2.70	3.454 (3)	136
C41—H41*B*⋯O4^v^	0.97	2.56	3.527 (3)	173

Symmetry codes: (i) 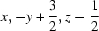; (ii) 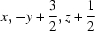; (iii) 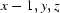; (iv) 
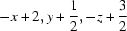
; (v) 
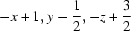
.

**Fig. 1.**
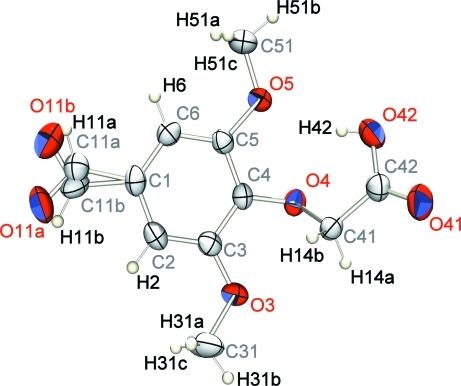
Molecular structure of the title compound with the numbering scheme. Displacement ellipsoids are drawn at the 50% probability level and H atoms are represented as spheres with an arbitrary radius.

**Fig. 2.**
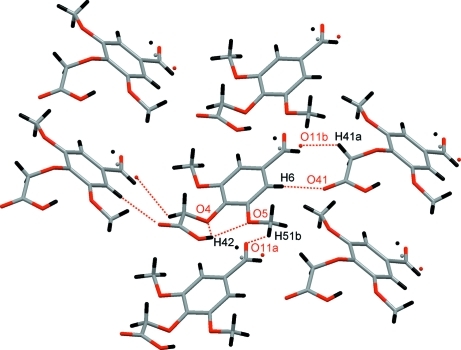
Corrugated sheets in the ac plane generated by weak intermolecular hydrogen bonds and the bifurcated intramolecular hydrogen bond.

**Fig. 3.**
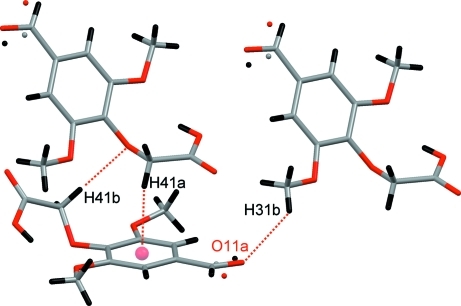
The two weak hydrogen bonds and the CH···π interaction responsible for the stacking of the sheets along the b axis.

## supplementary crystallographic information

### Comment

The title compound was synthesized as a precursor for asymmetric PPV-type [poly(*p*-phenylene vinylene)] oligomers. These compounds are promising candidates as the active materials in organic memories (Bandyopadhyay & Pal, 2003) and as nonlinear optical (NLO) materials with a high first-order hyperpolarizability (Chemla, 1987). Besides the condition that these oligomers should bear acceptor and donor substituents connected by a π-system, it is of critical importance for their usefulness as an NLO material and as a bistable organic memory material that the crystal packing is non-centrosymmetric. In particular, the compound should crystallize in a polar space group. To engineer the crystal packing in order to meet this criterion, it is necessary to introduce certain statistically well chosen synthons (Desiraju, 1997). Therefore, we opted for carboxylic acid functional groups (powerful hydrogen bond donors) in the basic structure of the organic semiconductors. To combine the electronic (A-π-D) and the structural (non-centrosymmetric space group) requirements, we used the Williamson ether synthesis to prepare the title compound as a building block for a PPV-based semiconductor bearing a carboxylic acid moiety.

The carboxylic acid moiety is found in the [*ap*,*ap*] conformation (Fig. 1): the carboxyl H atom points in the opposite direction of the carbonyl group and the O4—C41—C42—O41 torsion angle is -168.64 (19)°. The reason for this unexpected conformation is the presence of an intramolecular bifurcated hydrogen bond involving H42, O4 and O5 (Table 1 and Fig. 2). Indeed, Lide showed, based on microwave experiments on gaseous formic acid, that, in general, the *sp* conformer is more stable than the *ap* conformer by about 16 kJ mol^-1^ (Lide, 1964). The acetic acid chain is twisted out-of-plane by about 113° and the torsion angle O4—C41—C42—O42 is about 11°.

A sawtooth motif is generated along the *c* axis by the CH···O interaction between H6 and O41 (Fig. 2). Its effect is reinforced by H41A contacting O11B, the aldehyde O atom of the minor conformer. Perpendicular to these sawtooth ribbons (along the *a* axis) C51—H51B···O11A hydrogen bonds continue the structure (Fig. 2). These corrugated sheets are then stacked along the *b* axis by two weak CH···O hydrogen bonds and a CH···π interaction (Fig. 3): H31B contacts O11A, H41B contacts O4 and H41A contacts the centroid of the ring [C41—H41A···*Cg*^vi^, 2.68 Å, 144°, symm. code vi = 1 - *x*, 1/2 + *y*, 3/2 - *z*]. Finally, there is a modest π–π interaction [*Cg*···*Cg*^vii^, 4.133 (2) Å, 34.09°, symm. code vii = 1 - *x*, 2 - *y*, 1 - *z*].

### Experimental

Sodium hydroxide (5.0 g, 0.125 mol) in distilled water (20 ml) was added to a stirred solution of syringaldehyde (11.8 g, 0.065 mol) and iodoacetic acid (12.1 g, 0.065 mol) in distilled water (100 ml). The mixture was refluxed overnight and then poured into water (100 ml), which was then acidified using phosphoric acid. The mixture was left to cool in the refrigerator and the solvent was partially evaporated to precipitate the compound. The brown needle-like crystals were filtered off. The product changed colour to purple when it was exposed to air. The purple product was recrystallized from a 5:1 dichloromethane/acetone mixture to yield (54%) colourless crystals. ^1^H NMR (CDCl_3_, 400 MHz, TMS): δ 3.98 (s, 6H, H31 and H51), 4.69 (s, 2H, H41), 7.16 (s, 2H, H2 and H6), 9.89 (s, 1H, H11). ^13^C NMR (CDCl_3_, 100 MHz, TMS): δ 56.6 (C31 and C51), 70.3, (C41), 106.6 (C2 and C6), 132.9 (C1), 141.1 (C4), 152.6 (C3 and C5), 170.2 (C42), 190.5 (C11).

### Refinement

The aldehyde moiety proved to be orientationally disordered over two sites in a 79:21 ratio (refined values 0.785 (6):0.215 (6)). The structure was initially refined with the aldehyde group in one position and then the second site was located in the residual electron density map. For the two aldehyde functions, consisting of three atoms, O11A and O11B were restrained to have the similar anisotropic displacement parameters, and the C1—C11A/C1—C11B and C11A—O11A/C11B—O11B distances were restrained to be equal. C11A and C11B were constrained to posses identical anisotropic displacement parameters. H atoms H11A and H11B were placed on the geometrically calculated positions and refined as 'riding'.

### Crystal data

**Table:**

C_11_H_12_O_6_	*F*(000) = 504
*M**_r_* = 240.21	*D*_x_ = 1.446 Mg m^−^^3^
Monoclinic, *P*2_1_/*c*	Melting point: 415 K
Hall symbol: -P 2ybc	Mo *K*α radiation, λ = 0.71073 Å
*a* = 9.350 (2) Å	Cell parameters from 25 reflections
*b* = 7.416 (1) Å	θ = 9.0–14.2°
*c* = 17.374 (8) Å	µ = 0.12 mm^−^^1^
β = 113.67 (3)°	*T* = 293 K
*V* = 1103.4 (6) Å^3^	Block, colourless
*Z* = 4	0.27 × 0.24 × 0.15 mm

### Data collection

**Table:**

Enraf–Nonius MACH3 diffractometer	*R*_int_ = 0.030
Radiation source: fine-focus sealed tube	θ_max_ = 25.3°, θ_min_ = 2.4°
graphite	*h* = −11→11
ω/2θ scans	*k* = 0→8
4025 measured reflections	*l* = −20→20
2015 independent reflections	3 standard reflections every 60 min
1292 reflections with *I* > 2σ(*I*)	intensity decay: 1%

### Refinement

**Table:**

Refinement on *F*^2^	Secondary atom site location: difference Fourier map
Least-squares matrix: full	Hydrogen site location: inferred from neighbouring sites
*R*[*F*^2^ > 2σ(*F*^2^)] = 0.036	H-atom parameters constrained
*wR*(*F*^2^) = 0.093	*w* = 1/[σ^2^(*F*_o_^2^) + (0.0427*P*)^2^ + 0.1341*P*] where *P* = (*F*_o_^2^ + 2*F*_c_^2^)/3
*S* = 1.00	(Δ/σ)_max_ < 0.001
2015 reflections	Δρ_max_ = 0.15 e Å^−^^3^
171 parameters	Δρ_min_ = −0.15 e Å^−^^3^
8 restraints	Extinction correction: *SHELXL97* (Sheldrick, 2008), Fc^*^=kFc[1+0.001xFc^2^λ^3^/sin(2θ)]^-1/4^
Primary atom site location: structure-invariant direct methods	Extinction coefficient: 0.0086 (18)

### Special details

**Table:**

Geometry. All e.s.d.'s (except the e.s.d. in the dihedral angle between two l.s. planes) are estimated using the full covariance matrix. The cell e.s.d.'s are taken into account individually in the estimation of e.s.d.'s in distances, angles and torsion angles; correlations between e.s.d.'s in cell parameters are only used when they are defined by crystal symmetry. An approximate (isotropic) treatment of cell e.s.d.'s is used for estimating e.s.d.'s involving l.s. planes.
Refinement. Refinement of *F*^2^ against ALL reflections. The weighted *R*-factor *wR* and goodness of fit *S* are based on *F*^2^, conventional *R*-factors *R* are based on *F*, with *F* set to zero for negative *F*^2^. The threshold expression of *F*^2^ > σ(*F*^2^) is used only for calculating *R*-factors(gt) *etc*. and is not relevant to the choice of reflections for refinement. *R*-factors based on *F*^2^ are statistically about twice as large as those based on *F*, and *R*- factors based on ALL data will be even larger.

### Fractional atomic coordinates and isotropic or equivalent isotropic displacement parameters (Å^2^)

**Table:**

	*x*	*y*	*z*	*U*_iso_*/*U*_eq_	Occ. (<1)
O11A	0.8177 (2)	0.6876 (3)	0.52772 (13)	0.0677 (9)	0.785 (6)
C11A	0.6811 (5)	0.6988 (12)	0.5115 (3)	0.0490 (13)	0.785 (6)
H11A	0.6121	0.6482	0.4613	0.059*	0.785 (6)
C11B	0.720 (3)	0.712 (5)	0.5283 (16)	0.0490 (13)	0.215 (6)
H11B	0.8278	0.7181	0.5571	0.059*	0.215 (6)
O11B	0.6629 (10)	0.6437 (14)	0.4603 (6)	0.078 (3)	0.215 (6)
O4	0.39907 (13)	1.04247 (17)	0.70196 (7)	0.0376 (3)	
O5	0.22382 (14)	0.89227 (19)	0.55872 (8)	0.0449 (4)	
O3	0.71034 (14)	1.0208 (2)	0.76755 (8)	0.0478 (4)	
C6	0.4523 (2)	0.7946 (3)	0.53433 (11)	0.0388 (5)	
H6	0.3923	0.7439	0.4824	0.047*	
C2	0.7062 (2)	0.8612 (3)	0.64352 (11)	0.0414 (5)	
H2	0.8144	0.8545	0.6638	0.050*	
C5	0.38175 (19)	0.8774 (2)	0.58135 (10)	0.0345 (4)	
O42	0.13206 (15)	0.9328 (2)	0.70058 (9)	0.0604 (5)	
H42	0.1655	0.9578	0.6649	0.091*	
C4	0.47318 (19)	0.9529 (2)	0.65853 (10)	0.0334 (4)	
O41	0.22968 (17)	0.8754 (2)	0.83608 (9)	0.0623 (5)	
C1	0.6143 (2)	0.7884 (3)	0.56601 (11)	0.0392 (5)	
C41	0.4098 (2)	0.9607 (3)	0.77859 (11)	0.0380 (4)	
H41A	0.4634	1.0416	0.8252	0.046*	
H41B	0.4701	0.8502	0.7880	0.046*	
C3	0.6358 (2)	0.9442 (2)	0.69064 (11)	0.0369 (4)	
C42	0.2502 (2)	0.9191 (3)	0.77494 (12)	0.0429 (5)	
C51	0.1233 (2)	0.8060 (3)	0.48261 (12)	0.0551 (6)	
H51A	0.1374	0.8599	0.4359	0.083*	
H51B	0.0167	0.8202	0.4755	0.083*	
H51C	0.1484	0.6800	0.4853	0.083*	
C31	0.8750 (3)	0.9877 (4)	0.80939 (15)	0.0730 (7)	
H31A	0.8941	0.8602	0.8120	0.110*	
H31B	0.9128	1.0359	0.8653	0.110*	
H31C	0.9282	1.0449	0.7788	0.110*	

### Atomic displacement parameters (Å^2^)

**Table:**

	*U*^11^	*U*^22^	*U*^33^	*U*^12^	*U*^13^	*U*^23^
O11A	0.0510 (16)	0.0877 (17)	0.0786 (16)	0.0113 (11)	0.0409 (12)	0.0007 (12)
C11A	0.050 (3)	0.052 (2)	0.049 (3)	0.002 (3)	0.025 (3)	0.002 (2)
C11B	0.050 (3)	0.052 (2)	0.049 (3)	0.002 (3)	0.025 (3)	0.002 (2)
O11B	0.084 (6)	0.101 (7)	0.053 (6)	0.007 (5)	0.031 (5)	−0.019 (5)
O4	0.0424 (7)	0.0359 (7)	0.0372 (7)	0.0054 (6)	0.0188 (6)	0.0017 (6)
O5	0.0315 (7)	0.0590 (9)	0.0409 (7)	0.0027 (6)	0.0109 (6)	−0.0073 (6)
O3	0.0327 (7)	0.0590 (9)	0.0445 (8)	−0.0028 (6)	0.0080 (6)	−0.0074 (7)
C6	0.0424 (11)	0.0409 (11)	0.0330 (9)	0.0001 (9)	0.0149 (8)	0.0011 (9)
C2	0.0340 (10)	0.0435 (11)	0.0503 (11)	0.0039 (9)	0.0206 (9)	0.0078 (9)
C5	0.0327 (9)	0.0366 (10)	0.0354 (9)	0.0020 (8)	0.0148 (8)	0.0043 (8)
O42	0.0361 (7)	0.0922 (13)	0.0540 (9)	0.0028 (8)	0.0193 (7)	0.0085 (9)
C4	0.0364 (9)	0.0309 (10)	0.0375 (9)	0.0034 (8)	0.0196 (8)	0.0026 (8)
O41	0.0623 (9)	0.0806 (12)	0.0556 (9)	0.0015 (9)	0.0359 (8)	0.0100 (8)
C1	0.0452 (10)	0.0375 (11)	0.0438 (11)	0.0053 (9)	0.0271 (9)	0.0068 (9)
C41	0.0399 (10)	0.0419 (11)	0.0321 (9)	0.0023 (9)	0.0142 (8)	−0.0017 (8)
C3	0.0349 (9)	0.0359 (10)	0.0394 (10)	−0.0019 (8)	0.0144 (8)	0.0036 (9)
C42	0.0434 (11)	0.0454 (12)	0.0433 (11)	0.0047 (9)	0.0210 (9)	0.0012 (9)
C51	0.0376 (11)	0.0738 (16)	0.0470 (12)	−0.0074 (11)	0.0095 (9)	−0.0118 (11)
C31	0.0452 (13)	0.0778 (18)	0.0681 (15)	0.0078 (12)	−0.0066 (11)	−0.0103 (14)

### Geometric parameters (Å, °)

**Table:**

O11A—C11A	1.195 (5)	C2—C1	1.384 (3)
C11A—C1	1.485 (4)	C2—H2	0.9300
C11A—H11A	0.9300	C5—C4	1.386 (2)
C11B—O11B	1.196 (17)	O42—C42	1.324 (2)
C11B—C1	1.498 (16)	O42—H42	0.8200
C11B—H11B	0.9300	C4—C3	1.395 (2)
O4—C4	1.382 (2)	O41—C42	1.197 (2)
O4—C41	1.430 (2)	C41—C42	1.500 (3)
O5—C5	1.371 (2)	C41—H41A	0.9700
O5—C51	1.430 (2)	C41—H41B	0.9700
O3—C3	1.359 (2)	C51—H51A	0.9600
O3—C31	1.435 (3)	C51—H51B	0.9600
C6—C5	1.383 (2)	C51—H51C	0.9600
C6—C1	1.389 (3)	C31—H31A	0.9600
C6—H6	0.9300	C31—H31B	0.9600
C2—C3	1.383 (3)	C31—H31C	0.9600
			
O11A—C11A—C1	124.3 (3)	C6—C1—C11B	130.1 (9)
O11A—C11A—H11A	117.8	O4—C41—C42	110.56 (15)
C1—C11A—H11A	117.8	O4—C41—H41A	109.5
O11B—C11B—C1	118.8 (19)	C42—C41—H41A	109.5
O11B—C11B—H11B	120.6	O4—C41—H41B	109.5
C1—C11B—H11B	120.6	C42—C41—H41B	109.5
C4—O4—C41	116.19 (13)	H41A—C41—H41B	108.1
C5—O5—C51	117.50 (14)	O3—C3—C2	126.16 (16)
C3—O3—C31	116.68 (16)	O3—C3—C4	114.84 (16)
C5—C6—C1	118.91 (17)	C2—C3—C4	119.00 (16)
C5—C6—H6	120.5	O41—C42—O42	121.25 (19)
C1—C6—H6	120.5	O41—C42—C41	121.93 (18)
C3—C2—C1	119.53 (17)	O42—C42—C41	116.83 (16)
C3—C2—H2	120.2	O5—C51—H51A	109.5
C1—C2—H2	120.2	O5—C51—H51B	109.5
O5—C5—C6	125.46 (16)	H51A—C51—H51B	109.5
O5—C5—C4	114.83 (15)	O5—C51—H51C	109.5
C6—C5—C4	119.71 (15)	H51A—C51—H51C	109.5
C42—O42—H42	109.5	H51B—C51—H51C	109.5
O4—C4—C5	118.19 (15)	O3—C31—H31A	109.5
O4—C4—C3	120.58 (16)	O3—C31—H31B	109.5
C5—C4—C3	121.20 (16)	H31A—C31—H31B	109.5
C2—C1—C6	121.65 (17)	O3—C31—H31C	109.5
C2—C1—C11A	122.7 (2)	H31A—C31—H31C	109.5
C6—C1—C11A	115.7 (2)	H31B—C31—H31C	109.5
C2—C1—C11B	108.2 (9)		
			
C51—O5—C5—C6	3.9 (3)	O11A—C11A—C1—C6	177.2 (6)
C51—O5—C5—C4	−175.66 (17)	O11A—C11A—C1—C11B	0(7)
C1—C6—C5—O5	−179.30 (17)	O11B—C11B—C1—C2	−179 (3)
C1—C6—C5—C4	0.2 (3)	O11B—C11B—C1—C6	0(4)
C41—O4—C4—C5	112.81 (18)	O11B—C11B—C1—C11A	3(4)
C41—O4—C4—C3	−69.2 (2)	C4—O4—C41—C42	−120.89 (17)
O5—C5—C4—O4	−3.6 (2)	C31—O3—C3—C2	−10.5 (3)
C6—C5—C4—O4	176.83 (16)	C31—O3—C3—C4	169.40 (19)
O5—C5—C4—C3	178.46 (16)	C1—C2—C3—O3	179.70 (17)
C6—C5—C4—C3	−1.1 (3)	C1—C2—C3—C4	−0.2 (3)
C3—C2—C1—C6	−0.7 (3)	O4—C4—C3—O3	3.3 (2)
C3—C2—C1—C11A	179.4 (5)	C5—C4—C3—O3	−178.83 (17)
C3—C2—C1—C11B	178.6 (17)	O4—C4—C3—C2	−176.79 (17)
C5—C6—C1—C2	0.6 (3)	C5—C4—C3—C2	1.1 (3)
C5—C6—C1—C11A	−179.4 (4)	O4—C41—C42—O41	−168.63 (19)
C5—C6—C1—C11B	−178 (2)	O4—C41—C42—O42	11.4 (2)
O11A—C11A—C1—C2	−2.9 (10)		

### Hydrogen-bond geometry (Å, °)

**Table:**

*D*—H···*A*	*D*—H	H···*A*	*D*···*A*	*D*—H···*A*
O42—H42···O4	0.82	2.11	2.617 (2)	120
O42—H42···O5	0.82	2.18	2.932 (2)	153
C6—H6···O41^i^	0.93	2.54	3.468 (3)	176
C41—H41A···O11B^ii^	0.97	2.71	3.189 (9)	111
C51—H51B···O11A^iii^	0.96	2.57	3.369 (3)	141
C31—H31B···O11A^iv^	0.96	2.70	3.454 (3)	136
C41—H41B···O4^v^	0.97	2.56	3.527 (3)	173

Symmetry codes: (i) *x*, −*y*+3/2, *z*−1/2; (ii) *x*, −*y*+3/2, *z*+1/2; (iii) *x*−1, *y*, *z*; (iv) −*x*+2, *y*+1/2, −*z*+3/2; (v) −*x*+1, *y*−1/2, −*z*+3/2.

## References

[bb1] Bandyopadhyay, A. & Pal, A. J. (2003). *Appl. Phys. Lett.* **82**, 1215–1217.

[bb2] Chemla, D. S. (1987). *Nonlinear Optical Properties of Organic Molecules and Crystals* Boston: Academic Press.

[bb3] Desiraju, G. R. (1997). *J. Chem. Soc. Chem. Commun.* pp. 1475–1482.

[bb4] Enraf–Nonius (1994). *CAD-4 EXPRESS* Enraf–Nonius, Delft, The Netherlands.

[bb5] Farrugia, L. J. (1997). *J. Appl. Cryst.* **30**, 565.

[bb6] Farrugia, L. J. (1999). *J. Appl. Cryst.* **32**, 837–838.

[bb7] Harms, K. & Wocadlo, S. (1996). *XCAD4* University of Marburg, Germany.

[bb8] Leiserowitz, L. (1976). *Acta Cryst.* B**32**, 775–802.

[bb9] Lide, D. R. Jr (1964). *Annu. Rev. Phys. Chem.* **15**, 225–250.

[bb10] Lundquist, K., Stomberg, R. & von Unge, S. (1987). *Acta Chem. Scand. B*, **41**, 499–510.

[bb11] Sheldrick, G. M. (2008). *Acta Cryst.* A**64**, 112–122. 10.1107/S0108767307043930 18156677

[bb12] Spek, A. L. (2009). *Acta Cryst.* D**65**, 148–155. 10.1107/S090744490804362X PMC2631630 19171970

[bb13] Steiner, T. (2002). *Angew. Chem. Int. Ed.* **41**, 48–76.

